# Protocol for gaze estimation using facial orientation in marmosets during a social preference test

**DOI:** 10.1016/j.xpro.2026.104556

**Published:** 2026-05-07

**Authors:** Zefeng Wei, Maria Harbers, Atsu Aiba

**Affiliations:** 1 Laboratory of Animal Resources, Center for Disease Biology and Integrative Medicine, Graduate School of Medicine, The University of Tokyo, Tokyo, Japan

**Keywords:** Neuroscience, Cognitive Neuroscience, Behavior

## Abstract

Here, we present an open-source platform for evaluating social preference in common marmosets using image-based visual stimuli and gaze estimation. We describe the procedures for setting up the behavioral test, training marmosets to perform the test, and analyzing the resulting data with the provided code. This platform enables simple and noninvasive assessment of social attention toward social visual stimuli.

For complete details on the use and execution of this protocol, please refer to Harbers et al.^1^

## Highlights

• Steps for setting up the visual stimulus presentation system in marmosets
• Guidance on training marmosets to perform the social preference test
• Code for gaze estimation to evaluate responses to the visual stimuli

## Abstract (editor highlights)

Publisher’s note: Undertaking any experimental protocol requires adherence to local institutional guidelines for laboratory safety and ethics.

## Graphical abstract

**Figure:**
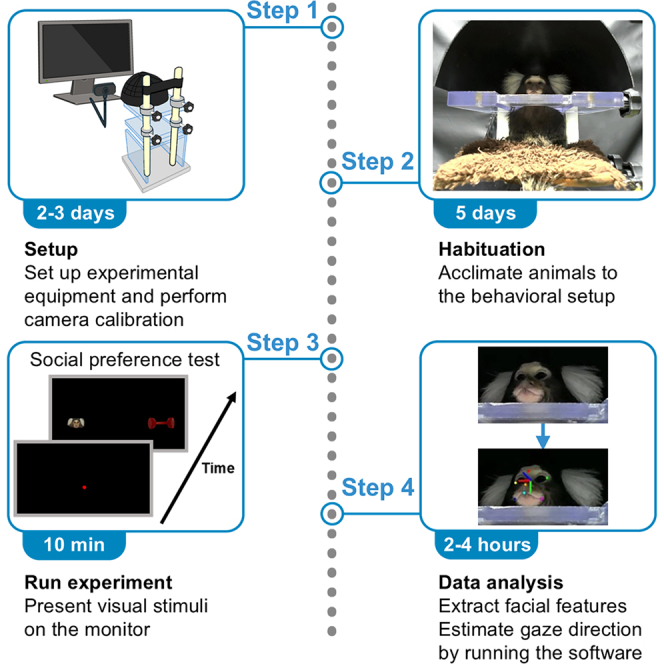

## Before you begin

Assessment of gaze direction provides a powerful approach for quantifying visual attention and behavioral responses in primates. Eye-tracking paradigms using image- and video-based stimuli have been widely employed in humans to quantify differences in gaze patterns, including responses to social stimuli.^2^ The common marmoset (*Callithrix jacchus*) has emerged as a valuable non-human primate model for studying social behavior and neurodevelopmental disorders.^3^ However, most eye-tracking techniques in marmosets require invasive head fixation, which limits their broader applicability, particularly in genetically engineered disease models.^4^^,^^5^^,^^6^ Here, we describe a noninvasive platform for assessing attention to visual stimuli in marmosets through gaze estimation based on facial orientation and demonstrate its application in evaluating social preference. We provide step-by-step procedures for setting up the behavioral test, training the animals, and analyzing the data. All codes, training guidelines, and setup instructions are available in an open repository to enhance reproducibility and accessibility for the broader research community. We also provide a sample dataset in the repository so that users can perform a test run of the system in their own environments. This platform enables quantitative and straightforward estimation of gaze direction and can be applied not only to the assessment of social preference but also to the evaluation of gaze responses to diverse visual stimuli tailored to specific research objectives.

### Innovation

This protocol presents a simple and noninvasive system for assessing social preference in marmosets. Unlike conventional methods that require head fixation, this approach estimates gaze direction from facial orientation during visual stimulus presentation. The system enables efficient and quantitative evaluation of visual attention, facilitating comparative and translational studies of social cognition and neurodevelopmental disorders.

### Institutional permissions

All experiments were reviewed and approved by the Institutional Animal Care and Use Committee of the University of Tokyo (permission number M-P21–033 and A2023M051-03) and conducted following the “Manual for Animal Experiment of the University of Tokyo” and “Guidelines for the Care and Use of Nonhuman Primates in Neuroscience Research” of The Japan Neuroscience Society. The study was also carried out in compliance with the ARRIVE (Animal Research: Reporting of In Vivo Experiments) guidelines for animals.

**Figure 1:**
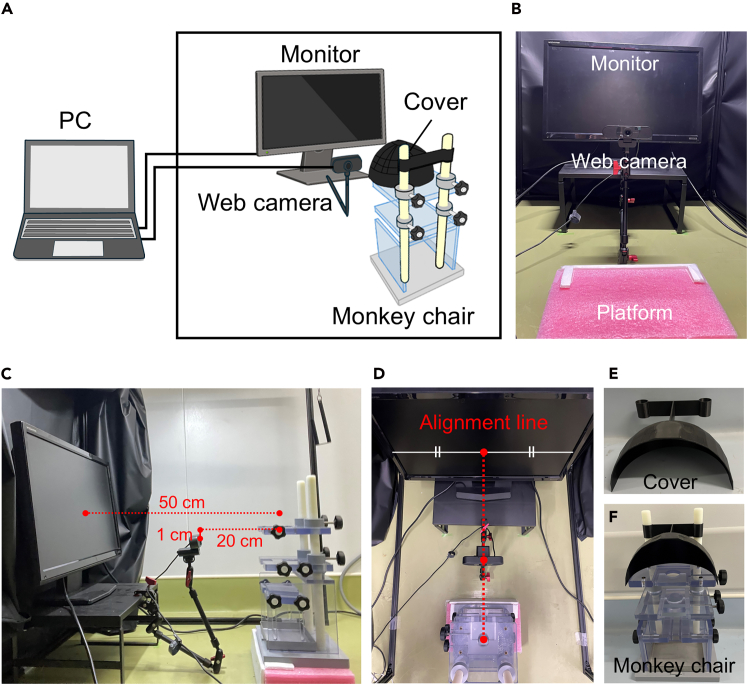
Setup of the experimental room (A) Schematic illustration of the experimental room. (B–D) Layout of the monitor, web camera, and platform. Rear view of the setup (B). Side view showing the distance between the monitor and chair (50 cm), the distance between the camera and chair (20 cm), and the relative height of the camera and the head-fixation holder of the chair (camera positioned 1 cm below) (C). Top view showing the alignment of the monitor, camera, and center of the chair (D). (E) A cover printed using a 3D printer. (F) A monkey chair equipped with the printed cover.

### Setup of the experimental room and video recording

**Timing: 2–3 days**

1. Set up the experimental room (800 mm × 900 mm × 1000 mm), covered with blackout curtains and equipped with a monitor, web camera, and platform for the monkey chair (Figures 1A–1D).
  a. Place the monitor on a monitor stand (390 × 250 × 185 mm).
  b. Position the platform so that the distance between the center of the monitor and the center of the head-fixation component of the monkey chair is 50 cm (Figures 1C and 1D).
    i. Place the monkey chair at the horizontal center of the monitor.
    ii. Adjust the platform height so that the visual stimuli are presented at the marmoset’s eye level when the animal is seated.
  c. Attach a camera to the monitor stand using a holder (Figure 1C).
    i. Attach a camera holder to the monitor stand and mount the web camera 20 cm away from the animal.
    ii. Position the camera 1 cm below the head-fixation component of the chair so that it does not obstruct the animal’s view of the monitor.
      **CRITICAL:** Align the centers of the chair, monitor, and camera using physical measurements to ensure proper horizontal alignment (Figure 1D). Verify alignment by confirming that a visual stimulus presented at the center of the monitor appears centered relative to the monkey chair, and that the marmoset’s face appears at the center of the image captured by the web camera. Mark the platform to ensure that the position of the monkey chair remains consistent across experiments.
  d. Connect the monitor and web camera to a recording PC located outside the experimental room.
  e. Set the recording PC desktop to a black background and configure the monitor as an extended display.
    ***Note:*** Minimum hardware specifications and setup constraints are as follows.
    Camera: The camera should have a resolution of 1280 × 720 pixels and a frame rate of 30 fps. A lens with a 70–90° field of view is recommended, and the camera should be positioned at a distance from the chair to capture the marmoset’s entire face. Cameras with substantial geometric distortion, such as some wide-angle models, should be avoided.
    Monitor: The appropriate monitor size depends on the visual stimuli and the distance between the monitor and the marmoset. A refresh rate of 60 Hz is sufficient when static images are used as stimuli. To avoid excessive relative viewing angles, the monitor should be placed at least 50 cm away from the marmoset. For longer distances, a monitor with a half-width of at least distance × tan(25°) is recommended to ensure sufficient space for stimulus placement.
    Illumination: The illumination inside the monkey chair cover should be maintained at approximately 20 lux to ensure that the marmoset’s face is clearly visible.
2. Prepare a cover to direct the marmoset’s attention toward the monitor (Figures 1E and 1F).
  a. Using the 3D stereolithography files (HeadCover.stl) provided in our GitHub repository (https://github.com/weizefeng-animal-resource/MarmosetGazeEstimation), print the cover with a 3D printer.
    ***Note:*** The provided STL file is designed specifically for the monkey chair model used in this study. Use the same chair model to ensure proper compatibility.
  b. Attach the printed cover to the monkey chair.

### Establishment of the software environment

**Timing: 1–2 h**

3. Set up the working environment on both the analysis PC and the recording PC.
  a. Install the Anaconda Python Platform (https://www.anaconda.com/products/distribution) on the analysis PC.
  b. Create a conda environment to run the Python script and DeepLabCut.
    i. Download DEEPLABCUT.yml file from the project GitHub repository (https://github.com/weizefeng-animal-resource/MarmosetGazeEstimation).
    ii. Open the Anaconda Command Prompt and install the environment using the following commands.
      cdpath/to/the/folder/containing/DEEPLABCUT.yml
      conda env create -f DEEPLABCUT.yml
  c. After the environment is successfully created, activate the new ‘DEEPLABCUT’ environment using the following command.
    conda activate DEEPLABCUT
  d. Set up the working directory for the analysis PC.
    i. Download analysis.py from the same repository.
    ii. Move the Python script to a new empty directory.
    iii. Use this directory as the working directory on the analysis PC.
    iv. Run the following commands in the Anaconda Command Prompt.
      cd path/to/the/working/directory
      python analysis.py -t createDirs
    v. Confirm that the required directory structure has been created in the working directory.
  e. Set up the working directory for the recording PC.
    i. Repeat steps a to c on the recording PC (use EXPERIMENT.yml instead of DEEPLABCUT.yml in step b).
    ii. Download recording.py from the same repository.
    iii. Move the Python script to a new empty directory.
    iv. Confirm that the required directory structure has been created in the working directory.
  f. Activate the ‘EXPERIMENT’ environment and run the following commands in the Anaconda Command Prompt to generate the necessary directories.
    cd path/to/the/working/directory
    python recording.py -t createDirs

### Camera calibration

**Timing: 1–2 h**

***Note:*** Estimating gaze points requires the intrinsic parameters of the web camera and the relative positions of the monitor and web camera, which can be obtained by calibration using a video camera.

**Figure 2:**
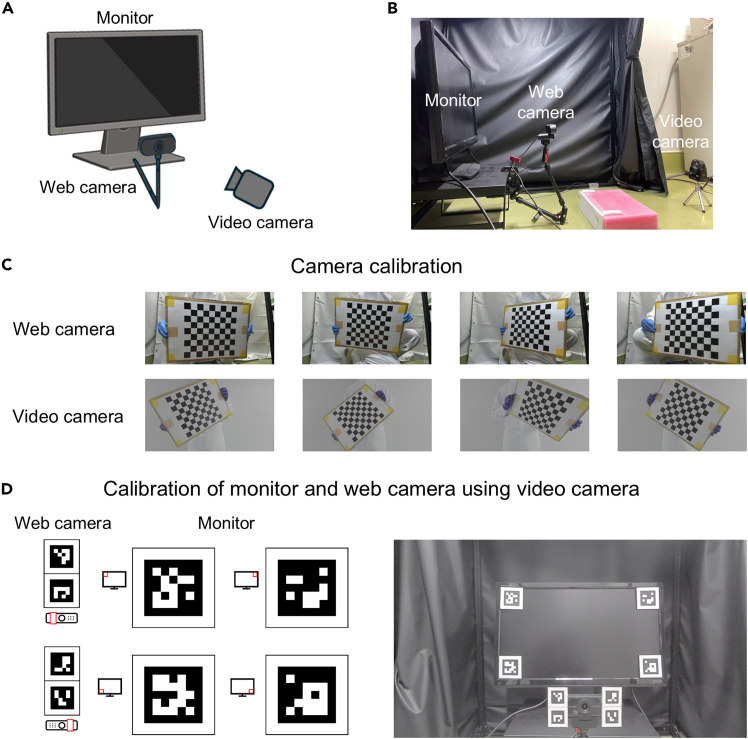
Camera calibration procedure (A) Schematic illustration of the calibration procedure. (B) Relative positions of the monitor, web camera, and video camera. (C) Calibration of the web camera and the video camera using a chessboard. Top: images captured by the web camera. Bottom: images captured by the video camera. (D) Calibration of the monitor and the web camera with reference to the video camera. Left: Positions where ArUco markers are attached to the web camera and the monitor. Right: Image of the web camera and monitor with the ArUco markers attached, captured by the video camera.

4. Position the video camera behind the monkey-chair platform and orient it so that both the monitor and the web camera are visible within the field of view (Figures 2A and 2B).

**Figure 3:**
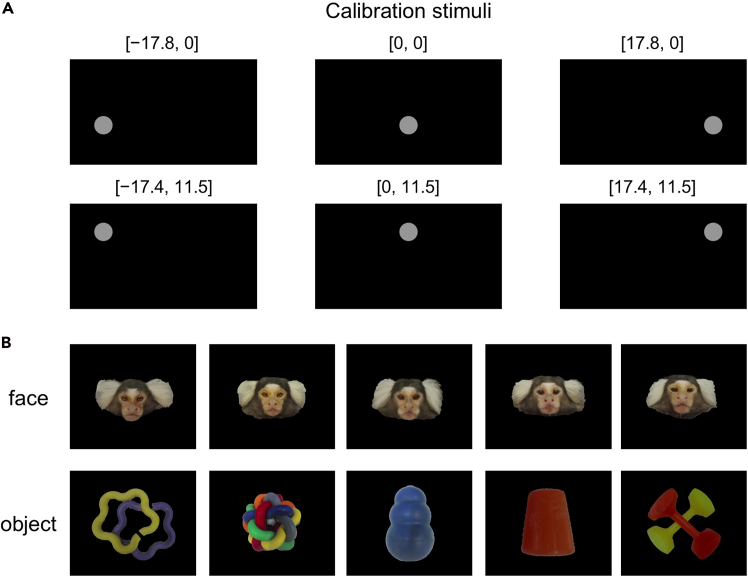
Visual stimuli (A) Six locations used for calibration stimuli arranged in a 3 × 2 grid. The top row shows the positions where the cue and either the face or object stimuli are presented during the test, and the bottom row shows locations used to calibrate points above the stimulus presentation positions (angles in degrees: [horizontal, vertical]). (B) Examples of face and object images used in the social preference test.

**Figure 4:**
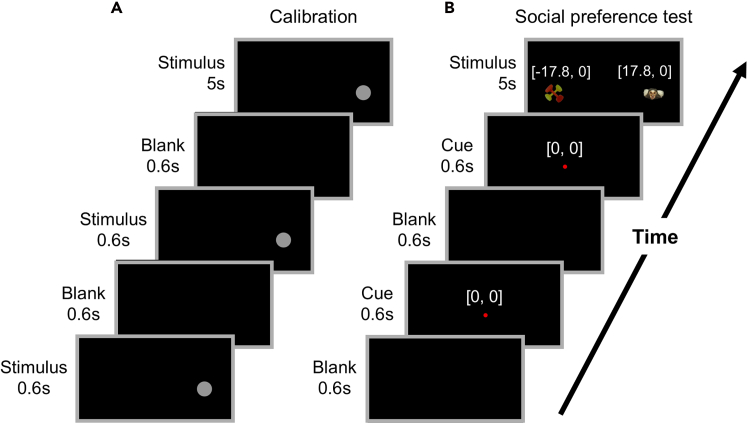
Visual stimulus presentation procedures (A and B) Procedures for presenting a calibration stimulus (A) and a visual stimulus for the social preference test (B). Angles of the cue and the left and right stimulus presentations are indicated in (B) (angles in degrees: [horizontal, vertical]).
5. Calibrate both the web camera and the video camera (Figure 2C).
  a. Prepare a chessboard.
    i. Download Chessboard.pdf provided in the GitHub repository.
    ii. Print it on A3-size paper.
    iii. Mount the printed chessboard pattern onto a flat, rigid surface such as a piece of cardboard.
  b. Calibrate the **web camera.**
    i. Using the **web camera**, capture 10-15 images of the printed chessboard from different viewing angles.
      **CRITICAL:** Ensure that the lighting is bright and uniform across the entire field of view. Poor illumination, uneven lighting, or reflections from nearby direct light sources may interfere with the detection of the chessboard pattern.
    ii. Move the captured images into the **Camera1** folder in your working directory.
  c. Open the Anaconda Command Prompt and execute the camera calibration by running the following commands.
    ***Note:*** When the calibration completes successfully, a new file named camera1.pickle will be created in the working directory.
    conda activate DEEPLABCUT
    cd path/to/your/working/directory
    python analysis.py -t calCamera1
  d. Calibrate the **video camera.**
    i. Using the **video camera**, capture 10-15 images of the printed chessboard from different viewing angles.
    ii. Move the captured images into the **Camera2** folder, and run the following command.
      python analysis.py -t calCamera2
      ***Note:*** If this process completes successfully, a new file named camera2.pickle will be created in your working directory.
6. Estimate the relative position of the monitor and web camera (Figure 2D).
  a. Prepare ArUco markers.
    i. Download ArUcoMarkers.pdf from the repository.
    ii. Print it at the original scale on A4-size paper.
    iii. Cut out each marker along its black border and mount the printed markers onto a flat, rigid surface such as a piece of cardboard.
      **CRITICAL:** Measure the edge length of the black marker regions. The larger markers for the monitor should have an edge length of 4.9 cm, whereas the smaller markers for the web camera should have an edge length of 2.0 cm.
  b. Attach the ArUco markers to the four corners of the monitor and to both sides of the web camera.
    **CRITICAL:** Ensure all ArUco markers are placed in the correct positions.
  c. Using the **video camera**, capture an overview image of the experimental setup, ensuring that all markers are clearly visible.
    **CRITICAL:** As before, ensure that the lighting is bright and uniform across the entire field of view. Poor illumination, uneven lighting, or reflections from nearby direct light sources may interfere with the detection of the ArUco markers.
  d. Determine the positional relationship between the monitor and the web camera.
    i. Move the captured image into the **Setup** folder and run the following command.
      python analysis.py -t locateSetup
    ii. Confirm that a file named “setup.pickle” and two images (“ArUco_marker_0.png” and “ArUco_marker_4.png”) have been generated in the working directory.
    iii. Verify that the ArUco markers are correctly detected and properly aligned in these images.
      ***Note:*** If an inappropriate image (e.g. a blurred or low-contrast image) is used, the process will be interrupted with an error message. Users can refer to the representative examples of both appropriate and inappropriate images (“setup_image_fine.png” and “setup_image_blurred.png”) provided in our repository.
  e. Maintain the position of the monitor and the web camera after this step.
    ***Note:*** If their relative positions are altered, backup the current setup.pickle for use with previously collected data, then repeat steps b to d to generate a new setup configuration.

### Creation of visual stimuli

**Timing: 1–2 h**

7. Create calibration stimuli.
  a. Install the AviUtl software (https://spring-fragrance.mints.ne.jp/aviutl/) on the analysis PC.
    ***Note:*** This software is compatible with Windows. Use other video editing software as needed.
  b. Create visual stimulus videos using AviUtl with a resolution of 1920 × 1080 and a frame rate of 25 fps.
    ***Note:*** The video resolution should match the monitor’s native resolution to avoid scaling artifacts.
  c. Prepare a circular visual stimulus with a diameter of 5.5° ± 0.2°.
  d. Arrange six predefined stimulus locations in a 3 × 2 grid on the screen (Figure 3A).
  e. Specify the stimulus positions in degrees [horizontal°, vertical°] as follows:
     [0°, 0°]: cue stimulus position during the test.
     [17.8°, 0°] and [−17.8°, 0°]: positions where face and object stimuli are presented during the test.
     [17.4°, 11.5°], [0°, 11.5°], and [−17.4°, 11.5°]: upper calibration points.
    ***Note:*** The horizontal and vertical angles listed above serve as an example of the stimulus layout used in this study and can be adjusted as needed depending on the positions of the stimuli used in the subsequent test.
  f. At each location, present the stimulus for 5 s following two brief presentations (Figure 4A). Present the stimuli in sequence at each location, so that each location receives two presentations in total.
  g. Export the video in MP4 format.
  h. Move the video to the working directory on the recording PC.
8. Create visual stimuli for the social preference test using AviUtl, with the same resolution, frame rate, and output format as the calibration stimuli.
  a. Prepare ten pairs of visual stimuli, each consisting of an unfamiliar marmoset’s neutral face and an object (Figure 3B).
  b. Create stimuli with an appropriate visual angle (in our study, 8.5° ± 3.5° in width and 6° ± 2° in height). Standardize all stimuli for size and total luminance.
  c. For each trial, present two central cue stimuli at [0°, 0°] for 0.6 s each, followed by a pair of face and object stimuli displayed for 5 s (Figure 4B).
  d. Position the face and object stimuli with a horizontal separation of 35.6°, aligned vertically with the cue stimulus, so that gaze shifts require head movements rather than eye movements.
  e. Repeat this procedure for 10 trials, counterbalancing the left–right positions of the face and object stimuli pairs across trials.
    ***Note:*** The angular separation should be set substantially greater than 10°, the range over which eye movement contributions to gaze shifts rapidly saturate in marmosets.^7^ Beyond this range, gaze shifts increasingly rely on head movements; thus, a sufficiently large horizontal separation ensures that shifts between stimuli require head movements rather than eye movements alone.
  f. Export the video in MP4 format.
  g. Move the video to the working directory on the recording PC.

### Frame delay verification

**Timing: 5 min**

9. Check for frame delay between visual stimulus presentation on the monitor and camera recording.
  ***Note:*** The following command records the stimulus displayed on the monitor using a web camera and detects frame delays based on luminance changes.
  a. Turn off the lights in the experimental room enclosed by blackout curtains so that the room is completely dark.
  b. Confirm that the camera and monitor are properly connected to the recording PC.
  c. Run the following code to measure the delay between monitor rendering and camera recording.
    ***Note:*** The same visual stimulus video used in the actual test should be used for this measurement. The code automatically detects the frame in which the luminance change from the flash appears in the camera recording.
    conda activate EXPERIMENT
    cd path/to/the/working/directory
    python recording.py -t detectDelays ∖
    -v name/of/the/video/for/stimulus/presentation.mp4
  d. Record the frame delay value displayed in the command line, as it will be used in subsequent analyses.

## Key resources table

**Table:** 

REAGENT or RESOURCE	SOURCE	IDENTIFIER
**Experimental models: Organisms/strains**		

Common marmosets (Callithrix jacchus), 19 males and 9 females, 1–11 years old	Breeding colony of The University of Tokyo	N/A

**Software and algorithms**		

DeepLabCut (Version 2.3.9)	Mathis et al.^8^	http://www.mackenziemathislab.org/deeplabcut
Python (Version 3.10.19)	Python	https://www.python.org/
Prism 9	GraphPad	RRID:SCR_002798
AviUtl (Version 1.10)	N/A	https://spring-fragrance.mints.ne.jp/aviutl/
Powerpoint	Microsoft	RRID:SCR_023631
Anaconda Python Platform v.25.3.1	Anaconda, Inc.	https://www.anaconda.com/
MarmosetGazeEstimation v.1.0.0	This paper	https://doi.org/10.5281/zenodo.19446478
Blender 4.2.0	Blender	https://www.blender.org/
Ultimaker Cura 5.10.0	Ultimaker	https://github.com/Ultimaker/Cura

**Other**		

Monitor	IODATA	Cat # LCD-MF234XPBR,
Monitor stand	ariamaru	Cat # h02-390160-bk
Web camera	EMEET	Cat # C960
Video camera	Zoom	Cat # Q2n-4K
Camera holder	JEBUTU	Cat # 3D2MSB-3DXQJ
Analysis PC	Lenovo	Cat # ThinkStation P920
Recording PC	MacBook Pro	Cat # MNEH3J/A
Monkey chair	Physio-Tech	N/A
Cover	3D-print	N/A
Black laboratory partition curtain	MORIMOTO KASEI	Cat # MADR-800×900×1000
3D printer	Creality	Cat # Ender-3 Pro

## Step-by-step method details

### Habituation

**Timing: 5 days**

This section describes the habituation of animals to the experimental room and the monkey chair. This step is critical because insufficient habituation can reduce the marmoset’s attention to the visual stimuli during the subsequent test. Train each animal to sit in the monkey chair in the experimental room for 10 min per day over 5 days.

1. Habituate the animals to the experimental room and monkey chair.
  a. Seat the marmoset in the monkey chair and attach the cover.
  b. Place the monkey chair on the platform in the experimental room and allow the animal to habituate for 10 min.
  c. If the marmoset is not facing forward, gently guide it to face the front. Troubleshooting 1.
  d. After 10 min, remove the monkey chair from the experimental room, provide a reward (sponge cake), and return the marmoset to the home cage.
  e. If the marmoset is not sufficiently habituated after 5 days, extend the training period by additional days. Habituate the marmoset until it remains seated facing forward for 10 min with no visual stimuli presented on the monitor.

***Note:*** In our cohort (n = 28), 16 animals had been previously habituated to the monkey chair in other experiments. Under these conditions, 27 animals completed habituation within 5 days, while one animal required 7 days. Animals with no prior experience with the monkey chair may require longer habituation periods.

### Social preference test

**Timing: 10 min**

The procedure of the test is described in this section. Calibration stimuli are first presented to estimate the marmoset’s gaze direction, followed by the presentation of visual stimuli used for the social preference test.

2. Start the experiment by opening the Anaconda Command Prompt and running the following commands. Select the calibration stimuli video file when prompted.

conda activate EXPERIMENT

cd path/to/the/working/directory

python recording.py -t run -c 0 ∖

-v name/of/the/video/for/calibration.mp4 ∖

-o name/for/the/output/file.mp4

***Note:*** The monitor will display only a black background until you press the ENTER key.

**CRITICAL:** Both the video file name and the output file name should end with the ‘.mp4’ extension. If the recording PC has an internal camera or is connected to multiple cameras, make sure that the value following ‘-c’ corresponds to the correct index of the web camera.

3. Seat the marmoset in the monkey chair and position it inside the experimental room.
  a. Seat the marmoset in the monkey chair and attach the cover.
  b. Place the monkey chair on the platform in the experimental room.

**CRITICAL:** Ensure that the marmoset is seated upright and facing forward. Improper positioning may lead to biased looking time toward one side while stimuli are presented.

4. Press the ENTER key to start the stimulus presentation and record the marmoset’s face.

**Figure 5:**
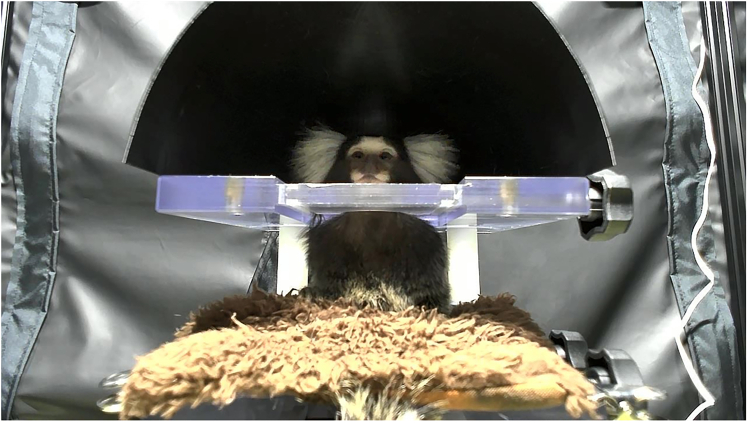
Web camera view

**CRITICAL:** The web camera view is displayed on the PC monitor (Figure 5). Confirm that the marmoset is facing the monitor before pressing the Enter key to begin stimulus presentation.

***Note:*** If necessary, press the ESC key to interrupt the experiment.

***Note:*** If a frame delay is detected in the frame delay verification section, note that the recorded data reflects this delay. Troubleshooting 2

5. After the video finishes, press the ENTER key to terminate the program.
6. Confirm that a new video file has been generated in the Results folder using the output name specified in step 2.
7. After the calibration stimuli are completed, allow a 90 s interval and provide a reward manually to the animal.
8. To perform the next experiment, run the following commands. Select the social preference test stimuli for the video file.

python recording.py -t run -c 0 ∖

-v name/of/the/video/for/stimulus/presentation.mp4 ∖

-o a/different/name/for/the/output/file.mp4

9. Repeat steps 4 and 5 to present the stimuli and begin recording.
10. After completing the stimulus presentation, remove the marmoset from the experimental room. Provide a reward and return it to the home cage.

### Gaze estimation

**Timing: 2–4 h**

This section describes the procedure for estimating gaze direction from facial landmarks using DeepLabCut. Videos of marmoset faces are first processed in DeepLabCut to label the specific facial landmarks required for gaze estimation. Calibration is then performed using selected frames to build a gaze estimation model for subsequent gaze point computation.

**Figure 6:**
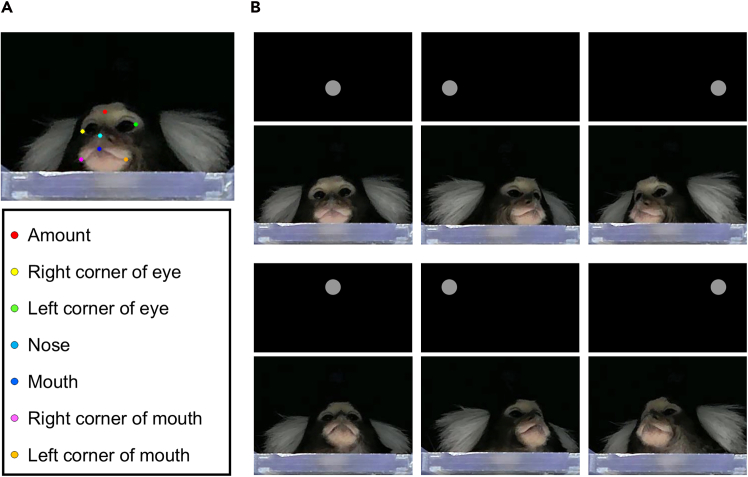
Procedure for gaze estimation (A) Facial landmarks labeled using DeepLabCut. (B) Example frames in which the marmoset was judged to be looking at each of the six calibration stimulus locations.

11. Detect the facial landmarks of marmosets from videos using DeepLabCut (Figure 6A).
  a. Launch the DeepLabCut GUI interface by running the following commands:
    conda activate DEEPLABCUT
    python -c “import deeplabcut; deeplabcut.launch_dlc()”
  b. Label the facial landmarks in the DeepLabCut GUI.
    i. Open the videos in DeepLabCut GUI and label the facial landmarks, including the amount, outer corners of both eyes, nose, center of the mouth, and both corners of mouth.
      ***Note:*** Keypoints that are occluded should be left unlabeled (e.g., when the marmoset rotates its head substantially and is not facing the monitor). Refer to the examples of keypoint labeling in the repository, including cases where all keypoints are visible (“labelling_example_visible.png”) and where some keypoints are occluded (“labelling_example_occluded.png”). Users can also refer to the pretrained network provided in the repository for additional examples of labeled images.
    ii. Train and evaluate the neural network until most of the landmark detection accuracies reach approximately 0.96, and estimate the positions of facial landmarks using the trained network. Troubleshooting 3.
      ***Note:*** Our trained network is provided in the repository, and an example video illustrating the tracking of facial landmarks is provided in Methods video S1. The network was trained on 192 manually labeled images (95% training fraction) for 500,000 iterations, yielding a training error of 1.06 pixels and a test error of 2.15 pixels. Detailed guidance can be found in the original repository of DeepLabCut (http://www.mackenziemathislab.org/deeplabcut).
      **CRITICAL:** The names of all facial landmarks must exactly match those shown in Figure 6A.
  c. Export the results in CSV format and move them into the DeepLabCutOutputs folder.
  d. Run the following command to estimate the gaze points.
    python analysis.py -t estimateGazePoints -a 0.96
    ***Note:*** Adjust the accuracy threshold (the value after '-a') depending on the performance of your DLC network.
  e. Confirm that the output has been generated in the Results folder in CSV format.
12. Calibrate the gaze point estimation system.
  a. Select the frames in which the marmosets were looking at calibration stimuli.
    ***Note:*** Select frames based on the moment when the marmoset faced the direction of the presented stimulus, and its head movement had stopped (Figure 6B).
  b. Edit the calibration_configs.csv file automatically generated in your working directory following the example provided in our GitHub repository (https://github.com/weizefeng-animal-resource/MarmosetGazeEstimation).
  c. Calibrate the gaze point estimation system by running the following commands.
    conda activate DEEPLABCUT
    cd path/to/your/working/directory
    python analysis.py -t calEstimator
  d. Confirm that a file named “calibrator.pickle” has been created in your working directory.
13. Calibrate the gaze points by running the following command. Confirm that the results have been generated in the CalibratedResults folder in CSV format. Troubleshooting 4.

python analysis.py -t calResults

**Optional:** To evaluate the quality of the results, you can generate a video annotated with facial landmarks and the 3D orientation of the marmoset’s face by running the following commands (Methods video S2). Move the recorded video and the played stimuli video to your working directory. The accuracy threshold (the value after “-a”) should match the value used in step 11-b. The annotated video will be generated in the **Annotated Videos** folder using the filename you specify.

python analysis.py -t annotateVideo ∖

-c name/of/the/file/in/CalibratedResults/folder.csv ∖

-m path/to/the/recorded/video.mp4 ∖

-s path/to/the/stimuli/video.mp4 ∖

-o name/of/the/output/file.mp4 ∖

-d (number of the frame delay) ∖

-a (the accuracy cutoff)

***Note:*** Based on previous findings in freely moving marmosets showing that eye movement contributions during gaze shifts are typically within approximately 10°, use this value as the tolerance window.^7^

14. Calculate the radius of the tolerance window around the stimulus by multiplying the screen distance (50 cm) by the tangent (10°) to determine whether the marmoset is looking at the visual stimulus.

## Expected outcomes

**Figure 7:**
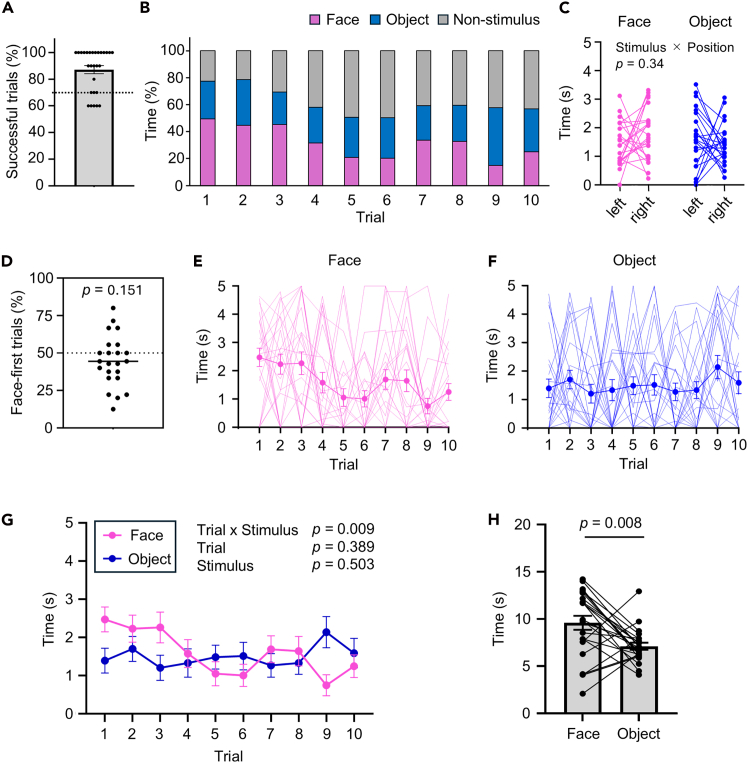
Results of the social preference test (A) Proportion of successful trials, defined as trials in which marmosets directed their gaze at least once toward the stimulus regions (face or object), across 10 trials. Data are shown as mean ± SEM. Each dot represents an individual. The dashed line indicates the criterion (70%). (B) Mean proportion of time spent looking at the face, object, and non-stimulus areas for each trial, averaged across individuals (magenta: face, cyan: object, gray: non-stimulus). (C) Gaze time for face and object stimuli depending on stimulus position (left or right). Each dot represents an individual. Statistical analysis was performed using a two-way repeated-measures ANOVA (stimulus category × position). (D) Proportion of trials in which the face stimulus was gazed at first. Each dot represents an individual marmoset, and the line indicates the median. A one-sample *t* test was used to compare the proportion with the chance level (50%). (E and F) Gaze time toward the face (E) and object (F) across trials. Thin lines represent individual animal data, while thick lines and error bars indicate the mean ± SEM across all subjects. (G) Comparison of the mean gaze time for the face and object shown in (E) and (F). A two-way repeated-measures ANOVA with stimulus category and trial as factors was used for statistical analysis. (H) Total gaze time directed toward the face and object stimuli across the first five trials. For each animal, the gaze time directed toward each stimulus category was summed across trials 1–5. Each dot represents an individual marmoset. Data are shown as mean ± SEM. A paired *t* test was used for statistical comparison between the two categories. Twenty-eight individuals (males: n = 19; females: n = 9) were tested. Analyses shown in panels (B)–(H) were performed using the 23 animals (males: n = 17; females: n = 6) that met the criterion shown in (A).

Successful implementation of this protocol enables quantitative assessment of marmosets’ visual preference for face compared to object. In a representative dataset, we tested 28 marmosets aged 1–11 years (male n = 19; female n = 9). The marmosets were maintained with ad libitum access to food and water. A success rate of ≥70% across 10 trials can be used as the inclusion criterion. In our case, 23 animals met this threshold and were included in subsequent analyses (Figure 7A; Troubleshooting 5).

During the test, face and object images are randomly presented on either the left or right side of the display. Marmosets’ gaze time can be quantified for stimulus (face and object) and non-stimulus areas in each trial (Figure 7B). No significant interaction is typically observed between stimulus category and position for gaze time, indicating that the category effect is independent of stimulus position (Figure 7C).

Preference between face and object stimuli can be evaluated by quantifying which stimulus was viewed first (Figure 7D) and the duration spent looking at each stimulus (Figures 7E–7G). The proportion of trials in which the face is viewed first is expected not to differ significantly from chance (Figure 7D). However, gaze time typically shows a significant Trial × Stimulus interaction (Figure 7G). Marmosets tend to spend longer looking at faces than objects, particularly during the first five trials (Figure 7H), reflecting a preference for social stimuli.

## Limitations

**Figure 8:**
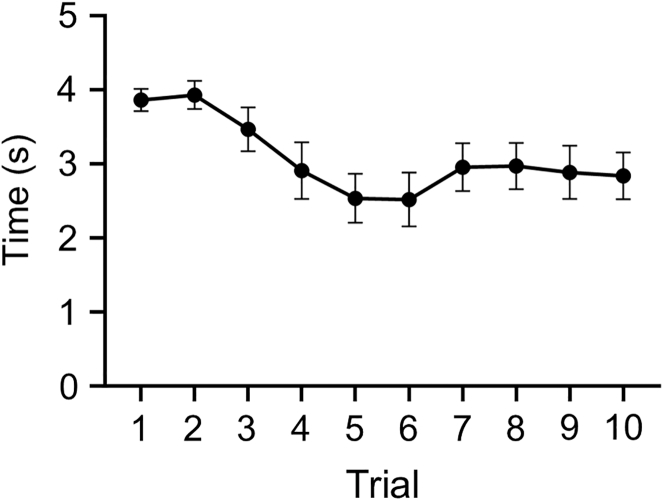
Total gaze time per trial in the social preference test Total gaze duration directed toward both the face and object stimuli in each trial. Data are shown as mean ± SEM.

This platform has two main limitations. First, detailed gaze patterns cannot be fully captured, as gaze estimation is based on facial orientation rather than direct eye tracking. Consequently, this approach cannot capture fine details of the face-scanning pattern. Second, marmosets’ attention to visual stimuli tends to decline over repeated trials, which reduces the time window during which stable social preference can be reliably evaluated (Figure 8; Troubleshooting 6).

## Troubleshooting

### Problem 1

The marmoset does not remain seated facing forward (Step-by-step method details, STEP 1).

### Potential solution

Adjust the height of the footrest attached to the monkey chair so that the marmoset can sit comfortably in a stable position. If the chair height is appropriate, attach a piece of soft cloth or other material to the front support plate (the part that holds the abdomen), because some marmosets feel more secure when they can grasp something while facing forward (as shown in Figure 5). In addition, provide a reward when the marmoset successfully sits facing forward to reinforce the desired posture.

### Problem 2

A frame delay is detected during frame delay verification (step-by-step method details, STEP 4).

### Potential solution

If a frame delay is detected, incorporate the measured delay into the gaze estimation process to ensure accurate temporal alignment during subsequent analysis.

### Problem 3

Facial landmarks are not tracked accurately (Step-by-step method details, STEP 11).

### Potential solution

Facial landmarks may be tracked inaccurately if the training dataset is insufficient or biased, if occluded keypoints are labeled incorrectly, or if lighting conditions differ between training and testing videos. Increasing the diversity of labeled frames, leaving occluded keypoints unlabeled, and maintaining consistent lighting conditions between recording sessions can improve tracking accuracy.

### Problem 4

Gaze estimation does not work properly (step-by-step method details, STEP 13).

### Potential solution

First, test the analysis pipeline using the demo dataset and the trained DeepLabCut network provided in the repository. If the pipeline runs successfully with these data, the issue is likely related to the experimental setup or the training of the DeepLabCut model. For the experimental setup, check the lighting conditions, camera angle, and animal positioning, and ensure that the recording environment matches the recommended setup described in this protocol. For issues related to network training, see problem 3.

### Problem 5

The marmoset does not look at the visual stimuli (Expected outcomes).

### Potential solution

If a marmoset fails to look at the visual stimuli in more than 3 trials out of the 10 trials, exclude that individual from the analysis.

### Problem 6

Marmosets’ attention declines across trials (Limitations).

### Potential solution

Reduce the number of trials per session to minimize loss of interest. If longer engagement is required, intermittent reward delivery (e.g., via an automated reward delivery system) may help maintain attention. The timing of reward delivery (e.g., during inter-trial intervals or around cue presentation) can be adjusted depending on the experimental objective.

## Resource availability

### Lead contact

Further information and requests for resources should be directed to and will be fulfilled by the lead contact, Atsu Aiba (aiba@m.u-tokyo.ac.jp).

### Technical contact

Technical questions on executing this protocol should be directed to and will be answered by the technical contact, Zefeng Wei (weizefeng@m.u-tokyo.ac.jp).

### Materials availability

This study did not generate new unique reagents.

### Data and code availability

The code used for analysis is available via GitHub (https://github.com/weizefeng-animal-resource/MarmosetGazeEstimation) and Zenodo (https://doi.org/10.5281/zenodo.19446478).

## Acknowledgments

This work is supported by Brain/MINDS (JP19dm0207071) and Brain/MINDS 2.0 (JP23wm0625001) from 10.13039/100009619AMED to A.A. and by the 10.13039/501100001691Japan Society for the Promotion of Science (JSPS) KAKENHI 24KJ0590 to M.H.

## Author contributions

Z.W.: methodology, software, visualization, and writing – original draft and editing; M.H.: methodology, investigation, visualization, writing – original draft and editing, and funding acquisition; A.A.: writing – review and editing and funding acquisition.

## Declaration of interests

The authors declare no competing interests.

## Supplemental information

Methods video S1. Representative video showing facial landmark tracking using our trained network, related to step 11

Methods video S2. Gaze estimation, related to step 13 Video recorded with the web camera, annotated with the facial landmarks and the 3D orientation of the marmoset’s face. The estimated gaze point was indicated by a red dot on the stimulus presented on the monitor.
